# Adoption of Preventive Behaviour Strategies and Public Perceptions About COVID-19 in Singapore

**DOI:** 10.34172/ijhpm.2020.199

**Published:** 2020-10-20

**Authors:** Semra Ozdemir, Sean Ng, Isha Chaudhry, Eric Andrew Finkelstein

**Affiliations:** Signature Programme in Health Services and Systems Research, Lien Centre for Palliative Care, Duke-NUS Medical School, Singapore, Singapore.

**Keywords:** COVID-19, Coronavirus, Preventive Behaviour Uptake, Public Perceptions, SARS-CoV-2, Singapore

## Abstract

**Background: **The unprecedented severity of coronavirus disease 2019 (COVID-19) constitutes a serious public health concern. However, adoption of COVID-19-related preventive behaviours remain relatively unknown. This study investigated predictors of preventive behaviours.

**Methods: **An analytical sample of 897 Singaporean adults who were quota sampled based on age, gender, and ethnicity were recruited through a web-enabled survey. Outcomes were adoption of, or increased frequency of preventive behaviours (avoiding social events; avoiding public transport; reducing time spent shopping and eating out; wearing a mask in public; avoiding hospitals/clinics; keeping children out of school, washing hands/using sanitisers; keeping surroundings clean; avoiding touching public surfaces; working from/studying at home). Public perceptions regarding COVID-19 (chances of getting COVID-19; perceived likelihood of COVID-19-related intensive care unit (ICU) admission; government trust; self-efficacy; perceived appropriateness of COVID-19 behaviours; response efficacy), anxiety, and demographic characteristics (age; ethnicity; marital status; education; chronic conditions; current living arrangements) were investigated as predictors of preventive behaviours adopted during COVID-19 in binomial and ordered logistic regressions.

**Results:** Though adoption of preventive behaviours among Singaporeans varied, it was, overall, high, and consistent with government recommendations. Nearly a quarter reported moderate to severe anxiety (General Anxiety Disorder 7-item – GAD-7 scores). Respondents who perceived higher COVID-19 risks, had higher government trust, higher self-efficacy, and perceived that others acted appropriately reported increased adoption/frequency of preventive measures. The strongest indicator of behavioural change was response efficacy. Respondents who were older, highly educated, anxious and married reported higher adoption/frequency of preventive measures.

**Conclusion:** To successfully influence appropriate preventive behaviours, public health messages should highlight response efficacy, increase self-efficacy, and promote trust in governmental response. Focus should be on demographic segments with low adoptions, such as younger individuals and those with low education.

## Background

**Key Messages**

**Implications for policy makers**

- Although adoption of individual preventive behaviours varied, adoption of the preventive measures among Singaporeans was, overall, high.
- The most widely adopted measures to avoid coronavirus disease 2019 (COVID-19) were avoiding or cancelling social events and reducing time spent shopping/eating out, consistent with the recommendations at the time.
- Response efficacy, self-efficacy, and government trust are important predictors of preventive behaviour adoption.

**Implications for the public**

Our findings underlined the importance of response efficacy, self-efficacy and government trust in motivating public adoption of preventive behaviours. Public health messages should focus on demographic segments with low adoptions, such as younger individuals and those with low education. These results will provide governments with a deeper understanding of the factors associated with preventive behaviour adoption during a pandemic, ensuring that the most appropriate interventions are put in place to best safeguard the health of the public.

The coronavirus disease 2019 (COVID-19) pandemic, originally detected in the Hubei province of China in late 2019, has since spread across the globe.^1-4^ The infection and fatality rates of COVID-19 are significantly higher than that of influenza – particularly in the elderly and individuals with pre-existing health conditions – making this outbreak a matter of grave public health concern.^5,6^ With pharmaceutical interventions for the prevention and treatment of COVID-19 still in development,^7^ non-pharmaceutical preventive measures such as personal protective equipment, promotion of preventive behaviours, and social quarantines are relied on to prevent spread.^8-10^ In Singapore, the setting of this study, these measures were conveyed to the public through extensive coverage by national news networks^11-13^ and citizen journalism sites.^14-16^ However, evidence on factors predicting the adoption of COVID-19 preventive behaviours, particularly in a Singaporean context, is limited. This underlines the importance of research focusing on a better understanding of the predictors of behavioural adoption in large-scale pandemics. Findings are expected to aid in the development of public health messages focused on increasing the adoption of preventive measures during future large-scale pandemics.

 Literature on past pandemics and current COVID-19 highlights the importance of public perceptions in influencing the adoption of preventive behaviours. When individuals reported higher self-efficacy^17,18^ (ie, they believed that they could prevent an infection) and higher risk perception,^19,20^ greater engagement in preventive behaviours was reported. Conversely, when people perceived the risk of COVID-19 to be exaggerated or disproportionate to the threat, preventive behaviours including hand-washing and social distancing decreased^21^. These results are consistent with the protection motivation theory (PMT) model, which proposes that individuals protect themselves based on several factors: perceived severity of event, probability of occurrence, perceived vulnerability, response efficacy, and self-efficacy.^22^

 Adoption of preventive behaviours, however, was not consistent across *all* protective behaviours. Measures that were perceived to have better response efficacy such as frequent hand washing and mask wearing had higher rates of adoption compared to those that were viewed as less efficacious (ie, working from home),^19^ suggesting that response efficacy may play an important role in the adoption of preventive behaviours. Public perception regarding the source of the preventive measures, in this case the respective governments, has also been found to predict protective behaviour adoption – government trust was positively correlated with adoption of preventive measures.^23^ In all, extant literature suggests that influencing public perception regarding pandemics is necessary to increase adoption of the preventive measures. However – while important – public perceptions of the respective pandemics were not the only factors associated with the adoption of precautionary measures.

 Generally, being female,^17,24^ older,^25^ married,^26^ having higher levels of education,^19,24^ and anxiety^27,28^ motivated positive behavioural changes during the COVID-19 pandemic. And while – to the best of our knowledge – there is little COVID-19 specific literature that examines the associations between positive behavioural changes and other demographic factors (ie, living arrangements, comorbidity) precedents from past pandemics demonstrate that living with young children^29^ or having comorbid diseases^30,31^ motivated preventive behaviours such as mask wearing and hand washing. And while there is little research done on the associations between living with elderly individuals and behavioural changes in pandemics, based on the increased risks of COVID-19 faced by the elderly, we expect that living with them would encourage behaviour change.

 Building on available literature, the aims of this study were to examine Singaporean public perceptions towards COVID-19 and to identify predictors of preventive behaviours in response to COVID-19. We hypothesised that perceived appropriateness of COVID-19 behaviours and high perceived COVID-19 risks, self-efficacy, response efficacy and government trust would be associated with adoption of preventive measures. We also hypothesized that being older, married, female, having higher education, higher anxiety, having pre-existing conditions and living with young children or the elderly would be associated with increased adoption of preventive behaviours. The survey was administered to an adult sample of Singapore’s general population during the initial stages of the COVID-19 pandemic. At the time of the survey, 683 (March 31, 2020) to 2631 (April 14, 2020) confirmed cases and 10 COVID-19 related deaths were reported in Singapore,^32^ significantly lower than in many countries (ie, America, China).^32^ It should also be noted the Singapore government implemented a circuit breaker, akin to a partial national lockdown, on seventh of April during our fieldwork. Restrictions at the time of the survey included, but were not limited to, travel advisories to infected countries, 14-day quarantines for people showing symptoms, cancelling of events with more than 250 people, social distancing of 1 metre, and requiring those with symptoms to wear masks^33,34^The analytical sample included only the respondents who completed the survey before the circuit breaker became effective.

## Methods

###  Study Settings

 A web-enabled survey was administrated by a market research company to their panel members between March 31, 2020 and April 14, 2020, and quota sampled based on Singapore’s census figures. Soft demographic quotas were also applied to age, gender and ethnicity with a +/- 5% error margin. The inclusion criteria of this study included being (1) aged 21 years old and above (the legal age of majority in Singapore) and (2) a resident of Singapore (ie, citizen, permanent resident, or work/dependent pass holder) at the time of the survey. A quota sample was taken based on gender, age, and ethnicity to ensure that the final sample would be nationally representative. Written informed consent was provided by all respondents. Respondents who completed the survey received small incentives via a point-based reward system paid by the survey company.

 The Singapore government implemented a circuit breaker, akin to a partial national lockdown, on seventh of April during our fieldwork. Restrictions included, but were not limited to mandatory working/studying from home arrangements, tighter workplace measures for essential workers, limited travel, controlled access to areas susceptible to crowds (ie, supermarkets), and restrictions on social gatherings with those outside one’s own household.^35^

###  Key Variables

####  Preventive Behaviours (Outcomes)

 As a measure of the preventive behaviours adopted during COVID-19, respondents answered “yes,” “no,” or “not applicable” to the question “in the past week, have you followed any of the strategies below (deliberately cancelled or avoided social events; avoided taking public transport; reduced time spent shopping; reduced frequency/duration spent eating out; wore a surgical mask in public; deliberately cancelled a doctor’s appointment or avoided hospitals or clinics; kept any of your children out of school) to reduce the risk of COVID-19 infection?” For all the activities listed above except for “kept any of your children out of school,” “no” and “not applicable” responses were merged under “no.” For the variable “kept any of your children out of school,” those who responded “not applicable” were excluded from the analysis since they may not have school-aged children.

 To examine preventive behaviours for which individuals could adopt increased frequency, respondents responded “same as usual,” “slightly more than usual” or “much more than usual” to the question “in the past week, how often did you follow any of the strategies below (washed hands with soap or use hand sanitisers; kept your surroundings clean; avoided touching public surfaces; worked from/studied at home) to reduce the risk of COVID-19 infection?”

####  Public Perception of COVID-19 in Singapore (Independent Variables)

 Three questions were used to gauge risk perceptions regarding the severity of COVID-19. First, respondents answered the question “what do you think your chances of getting COVID-19 are in comparison to regular flu (influenza)?” Response options included “much lower than the flu,” “slightly lower than the flu,” “same as the flu,” “slightly higher than the flu” or “much higher than the flu.” Additionally, respondents also responded to the perceived likelihood of intensive care unit (ICU) admission and the likelihood of dying upon getting COVID-19 on a 4-item Likert scale ranging from “very unlikely,” to “very likely.”

 To investigate the general public opinion regarding the COVID-19 outbreak in Singapore, respondents responded “strongly disagree,” “somewhat disagree,” “neither agree nor disagree,” “somewhat agree” or “strongly agree” to the statements: “I think the government will be successful in containing the spread of COVID-19,” “I think that if I am careful, I can reduce my chances of getting COVID-19,” and “I think people in Singapore are overly worried/paranoid about COVID-19.”

 Public perception regarding the effectiveness of preventive behaviours in reducing COVID-19 risk was examined through the question “how effective do you think the following strategies are: avoiding social events; avoiding public transportation; reducing time spent shopping; reducing frequency/duration spent eating out; wearing a mask in public avoiding hospitals or clinics; keeping children out of school; washing hands with soap or using hand sanitizers frequently; keeping your surroundings cleaner; avoid touching public surfaces; working from/studying at home. Response choices were “not effective,” “somewhat effective,” or “very effective.”

####  General Anxiety Disorder 7-item Scale (Independent Variable)

 Respondent anxiety was measured using the General Anxiety Disorder 7-item (GAD-7) Scale. The GAD-7 is a well-established and internal consistent scale (Cronbach’s α = 0.89 in an adult general population^36^). The scale contains a total of 7-items measured on 4-point Likert scale examining anxiety symptoms over the last 2 weeks (“not at all,” “several days,” “over half the days,” “nearly every day”).

####  Respondent Demographics (Independent Variables)

 Respondents were asked to report their age, gender, marital status, level of education, number of chronic health conditions and current living arrangements (ie, if they lived with an elderly person or young children). Although we did not have a specific hypothesis related to ethnicity, we included a dummy variable indicating Chinese ethnicity (vs. other race/ethnic groups) since Singapore consists of multiple ethnic groups, and some studies found that ethnicity was a predictor for adoption of preventive behavior.^37,38^

###  Statistical Analysis

 We first presented the descriptive summary statistics for demographic characteristics, anxiety, preventive behaviours adopted, and perceptions about COVID-19. Binomial logistic regression was performed for preventive behaviours that were adopted during COVID-19 (ie, deliberately cancelling or avoiding social events, avoiding public transportation, reducing time spent shopping, reducing frequency/duration spent eating out, wearing a surgical mask in public, deliberately cancelling a doctor’s appointment or avoiding hospitals or clinics, keeping children out of school). Ordered logistic regression was performed for preventive behaviours for which individuals could adopt increased frequency (ie, washing hands with soap or use hand sanitisers, keeping your surroundings clean, avoiding touching public surfaces, working from/studying at home).

 We first ran univariable regression with each independent variable (mentioned above) and included only those that were significant at 10% level in the multivariable models. In case of high correlation between two independent variables (*Pearson correlation coefficient ≥0.3*), one of the correlated variables were not included in the multivariable regressions. Independent variables included public perceptions regarding COVID-19: chances of getting COVID-19 in comparison to the regular flu (much higher or slightly higher vs. much lower, slightly lower, same), chances of being admitted to the ICU (very likely or somewhat likely vs. somewhat unlikely or very unlikely), chances of dying from COVID-19 (very likely or somewhat likely vs. somewhat unlikely or very unlikely), strong trust in COVID-19 related government policies (strongly agree vs. somewhat agree, neither agree/disagree, somewhat or strongly disagree), believing that they can reduce their chances of getting COVID-19 (strongly or somewhat agree vs. neither agree/disagree, somewhat or strongly disagree), thinking that the people in Singapore are overly worried/paranoid about COVID-19 (strongly agree or somewhat agree vs. neither agree/disagree, somewhat disagree, strongly disagree), and the effectiveness of the respective preventive behaviour (very effective vs. somewhat effective or not effective).

 We also included respondent demographics as part of independent variables: GAD-7 score, age categories 21-35, 36-50 (vs. over 50), married (vs. not), female (vs. male), higher education (university degree vs. lower), number of pre-existing chronic conditions, living with young children (vs. not), living with elderly (vs. not), and ethnicity (Chinese vs. non-Chinese).

 We used STATA 16.1 for all analyses.

## Results

###  Study Sample

 Of the 1338 quota-sampled participants, 98 participants terminated the survey and 223 participants had recorded partial responses. Out of remaining 1017 participants, 120 participants completed the survey after the national partial lockdown was implemented and were therefore excluded from the analysis. This left us a final analytical sample of 897 participants.

###  Respondent Characteristics

 About half of our respondents (47.6%) were female. The majority (84.2%) were Chinese with a median age of 40 years. There was a relatively uniform distribution of age groups, with 36.5% aged between 21 to 35 years old, 36.4% aged between 36 to 50 years old, and 28.1% aged > 50 years old. Overall, our sample is generally representative of the national population^39^ in terms of gender (51% female) and age (median age 41.1 years) but over-represents the Chinese ethnicity (74.3%). The majority were married (55.0%), university graduates (60.7%) and were living with elderly (≥65 years; 52.4%). 36.4% of respondents reported living with children below 12 years old. Over one-third of the respondents (34.7%) reported presence of at least one chronic condition with median (interquartile range, IQR) number of chronic conditions 2 (1, 3). 28.5% of the respondents reported mild anxiety (GAD 5-9) and about a quarter (23.8%) had moderate or severe anxiety (GAD score ≥10) (Table 1).

**Table 1:** Sample Characteristics and Risk Perceptions About COVID-19, N = 897

	**No. (%)**
**Respondent Characteristics**	
**Female**	427 (47.6)
**Age **(y)	
Mean (SD)	42 (12.8)
Median (IQR)	40 (32.0, 52.0)
> 50	252 (28.1)
21–35	327 (36.5)
36–50	318 (36.4)
**Ethnicity**	
Chinese	755 (84.2)
Malay	64 (7.1)
Indian + others	78 (8.7)
**Married**	493 (55.0)
**Education**	
Below university (no formal education, primary, secondary, vocational/ITE, JC/polytechnic/diploma/do not know/not sure)	353 (39.3)
University or above	544 (60.7)
**Living arrangement**	
Living with elderly (aged ≥65), yes	470 (52.4)
Living with young children (≤12), yes	327 (36.4)
**Chronic health conditions**	
At least one chronic health condition, yes	311 (34.7)
Median (IQR) for those with chronic conditions	2 (1.0, 3.0)
Mean (SD) for those with chronic conditions	2 (1.7)
**GAD-7 Score**	
Median (IQR)	5 (1.0, 9.0)
Mean (SD)	6 (5.5)
Mild anxiety (score: 5–9)	256 (28.5)
Moderate anxiety (score: 10–14)	151 (16.8)
Severe anxiety (score: ≥15)	63 (7.0)
**Public Perceptions of COVID-19 in Singapore**	
**Chances of getting COVID-19 compared to the regular Flu**	
Much lower than the flu, slightly lower than the flu, same as the flu	493 (55.0)
Slightly higher than the flu, much higher than the flu	404 (45.0)
**Perceived likelihood of COVID-19-related ICU admission**	
Very unlikely, somewhat unlikely	475 (53.0)
Very likely, somewhat likely	422 (47.0)
**Likelihood of Death from COVID-19**	
Very unlikely, somewhat unlikely	627 (69.9)
Very likely, somewhat likely	270 (30.1)
**I think the government will be successful in containing the spread of COVID-19**	
Neither agree/disagree, somewhat disagree, strongly disagree, somewhat agree	678 (75.6)
Strongly agree	219 (24.4)
**I think that if I am careful, I can reduce my chances of getting COVID-19**	
Neither agree/disagree, somewhat disagree, strongly disagree	218 (24.3)
Strongly agree, somewhat agree	679 (75.7)
**I think that people in Singapore are overly worried/paranoid about COVID-19**	
Neither agree/disagree, somewhat disagree, strongly disagree	574 (64.0)
Strongly agree, somewhat agree	323 (36.0)

Abbreviations: COVID-19, coronavirus disease 2019; IQR, interquartile range; SD, standard deviation; ITE, Institute of Technical Education; JC, junior college; ICU, intensive care unit. All figures are in percentages; Due to rounding, percentages may not add up to 100%. *IQR: inter-quartile range represents 25th and 75th percentile.

###  Public Perceptions of COVID-19 in Singapore

 Regarding perceptions of COVID-19 risk, 45.0% of respondents believed that they had “slightly higher” or “much higher” chances of getting COVID-19 compared to the regular flu (influenza) while 47.0% believed that they were either “somewhat likely” or “very likely” to be admitted to the ICU following COVID-19 infection. 30.1% of respondents felt that their likelihood of dying from COVID-19 was “very likely” or “somewhat likely” upon getting COVID-19 (Table 1).

 Nearly a quarter of the respondents (24.4%) indicated that they “strongly agreed” that the government would successfully contain the spread of COVID-19. The majority of respondents (75.7%) either “somewhat agreed” or “strongly agreed” that they would be able to reduce their chances of getting COVID-19 if they were careful. Only about a third of respondents (36.0%) “somewhat agreed” or “strongly agreed” that people in Singapore were overly worried or paranoid about COVID-19 (Table 1).

###  Adopted Preventive Behaviours

 The most adopted strategies to avoid COVID-19 in the past week were reducing time spent shopping (86.2%), reducing the frequency and duration spent eating out (86.1%) and cancelling or avoiding social events (82.8%). Keeping children out of school (43.2%) and cancelling a doctor’s appointment or avoiding hospitals and clinics (33.8%) were the least adopted measures (Table 2).

**Table 2:** Preventive Behaviour Strategies and Their Response Efficacy, N = 897

	**No. (%)**	**Response Efficacy (Very Effective), No. (%)**
**Adopted Preventive Behaviour Strategy, Yes**		
Cancelled or avoided social events	743 (82.8)	419 (46.7)
Avoided public transportation	395 (44.0)	275 (30.7)
Reduced the frequency/duration spent shopping	773 (86.2)	332 (37.0)
Reduced the frequency/duration spent eating out	772 (86.1)	327 (36.5)
Wore a surgical mask in public	516 (57.5)	330 (36.8)
Avoided hospitals or clinics	303 (33.8)	318 (35.5)
Kept children out of school^a^	184 (43.2)	119 (27.9)
**Preventive Behaviours That Could Be Adopted With Increased Frequency **		
Washing hands with soap or using hand sanitisers		
Same as usual	155 (17.3)	553 (61.7)
Slightly more than usual	389 (43.4)	
Much more than usual	353 (39.4)	
Keeping your surroundings cleaner		
Same as usual	258 (28.8)	407 (45.4)
Slightly more than usual	400 (44.6)	
Much more than usual	239 (26.6)	
Avoid touching public surfaces		
Same as usual	180 (20.1)	445 (49.6)
Slightly more than usual	367 (40.9)	
Much more than usual	350 (39.0)	
Working from/studying at home		
Same as usual	344 (38.4)	417 (46.5)
Slightly more than usual	262 (29.2)	
Much more than usual	291 (32.4)	

^a^N = 426 as” Not applicable” responses for the variable “Keeping children out of school” were removed as the respondents may not have school-aged children.

 To reduce risk of COVID-19 infection in the past week, respondents increased the frequency “much more than usual” of: washing hands with soap/using hand sanitisers (39.4%), keeping clean surroundings (26.6%), avoiding touching public areas (39.0%) and working/studying from home (32.4%) (Table 2).

 Washing of hands was considered to be the most effective strategy in reducing risk of getting COVID-19, with 61.7% considering it “very effective.” Keeping children out of school (27.9%) was perceived as the least effective strategy (Table 2).

###  Predictors of Preventive Behaviours Adopted During COVID-19

 Perceived risk of COVID-related death was highly correlated with perceived risk of COVID-related ICU admission (*Pearson correlation coefficient = 0.57*). Living with young children was highly correlated with being married (*Pearson correlation coefficient = 0.40*). Thus, perceived risk of COVID-related death and living with children were dropped from the multivariable analyses. Being female was not significant in univariable regressions (*P* >.10 for all) and was therefore excluded from the multivariable analyses.

####  Public Perceptions Regarding COVID-19 and Adoption of Preventive Behaviours

 Respondents who thought their chances of getting COVID-19 were higher in comparison to regular influenza had higher odds of cancelling social events and reducing time spent eating out. Similarly, perceived likelihood of COVID-19-related ICU admission was associated with higher odds of reducing time spent shopping and eating out and wearing masks in public. Government trust, however, did not significantly predict the adoption of any new preventive behaviours.

 Respondents who agreed that they could reduce their chances of getting COVID-19 if they were careful had higher odds of cancelling social events, reducing their time spent shopping and eating out. Those who agreed that Singaporeans were overly concerned or paranoid regarding COVID-19 had lower odds of reducing time spent shopping or eating out.

 Of note, higher perceived response efficacy was a significant predictor for adoption of all preventive behaviours (cancelling social events, avoiding public transport, reducing time spent shopping and eating out, wearing masks in public, avoiding hospitals and clinics and keeping children out of school). Response efficacy also had the largest odds ratios in predicting all preventive behaviours except cancelling social events (Table 3).

**Table 3:** Binomial Logistic Regressions on the Predictors of Adoption of Preventive Behaviours, N = 897 (Odds Ratios)

	**Cancel Social Events**	**Avoid Public Transport**	**Reduce Time Spent Shopping**	**Reduce Time Spent Eating out**	**Wearing Masks in Public**	**Avoiding Hospitals or Clinics**	Keeping Children out of School*
**Public Perceptions of COVID-19 in Singapore**							
Chances of getting COVID-19 compared to influenza (ref: Same/lower)							
Higher (slightly higher, much higher)	1.39	1.19	1.16	1.49	1.10	1.11	0.98
95% CI	* 0.95; 2.02*	*0.89; 1.59*	*0.77; 1.74*	*0.98; 2.27*	*0.82; 1.47*	*0.82; 1.50*	*0.63; 1.51*
*P* value	*0.09*	*0.24*	*0.49*	*0.06*	*0.52*	*0.49*	*0.92*
Likelihood of ICU admission if you get COVID-19 (ref: unlikely)							
Likely (very likely, somewhat likely)	1.31	1.25	1.63	1.77	1.47	1.10	1.07
95% CI	*0.89; 1.92*	*0.92; 1.68*	*1.06; 2.49*	*1.15; 2.73*	*1.09; 1.98*	*0.81; 1.50*	*0.68; 1.69*
*P* value	*0.17*	*0.15*	*0.03*	*0.01*	*0.01*	*0.53*	*0.76*
Government will be successful in containing the spread of COVID-19 (ref: disagree/no opinion)							
Agree (strongly agree, somewhat agree)	0.77	0.98	1.01	0.83	1.12	0.89	1.18
95% CI	*0.49; 1.21*	*0.69; 1.38*	*0.60; 1.70*	*0.50; 1.38*	*0.79; 1.59*	*0.62; 1.27*	*0.71; 1.95*
*P* value	*0.25*	*0.89*	*0.96*	*0.46*	*0.53*	*0.51*	*0.52*
I think that if I am careful, I can reduce my chances of getting COVID-19 (ref: disagree/no opinion)							
Agree (strongly agree, somewhat agree)	2.00	0.86	1.97	1.88	0.93	0.86	0.85
95% CI	*1.33; 3.01*	*0.61; 1.21*	*1.28; 3.05*	*1.20; 2.94*	*0.66; 1.31*	*0.61; 1.23*	*0.52; 1.40*
*P* value	*0.00*	*0.39*	*0.00*	*0.01*	*0.69*	*0.42*	*0.53*
I think that people in Singapore are overly worried/paranoid about COVID-19 (ref: disagree/no opinion)							
Agree (strongly agree, somewhat agree)	0.88	1.07	0.69	0.48	0.86	1.25	1.36
95% CI	*0.60; 1.29*	*0.79; 1.45*	*0.46; 1.05*	*0.32; 0.72*	*0.64; 1.16*	*0.91; 1.70*	*0.88; 2.10*
*P* value	*0.52*	*0.67*	*0.08*	*0.00*	*0.33*	*0.17*	*0.17*
Effectiveness of strategies in reducing risk of getting the COVID-19 virus (ref: not effective/somewhat effective)							
Very effective	1.87	3.55	4.22	3.28	3.44	2.55	4.19
95% CI	1.25; 2.78	2.59; 4.87	2.41; 7.38	1.94; 5.54	2.50; 4.72	1.87; 3.49	2.57; 6.84
*P* value	0.00	0.00	0.00	0.00	0.00	0.00	0.00
**Participant Demographics**							
GAD (anxiety)	1.05	1.04	1.03	1.03	1.04	1.09	1.08
95% CI	*1.01; 1.09*	*1.01; 1.07*	*0.99; 1.07*	*0.99; 1.07*	*1.01; 1.07*	*1.06; 1.12*	*1.03; 1.12*
*P* value	*0.02*	*0.01*	*0.19*	*0.21*	*0.01*	*0.00*	*0.00*
Age categories (ref: > 50 years old)							
21–35 years old	1.36	0.95	0.92	1.58	1.73	0.74	1.34
95% CI	*0.78; 2.39*	*0.62; 1.46*	*0.51; 1.66*	*0.86; 2.88*	*1.13; 2.64*	*0.48; 1.16*	*0.71; 2.53*
*P* value	*0.28*	*0.83*	*0.78*	*0.14*	*0.01*	*0.19*	*0.37*
36–50 years old	0.70	0.89	0.91	1.24	1.36	0.89	0.74
95% CI	*0.44; 1.11*	*0.61; 1.32*	*0.53; 1.56*	*0.72; 2.13*	*0.93; 1.98*	*0.60; 1.32*	*0.41; 1.31*
*P* value	*0.13*	*0.57*	*0.72*	*0.43*	*0.11*	*0.55*	*0.30*
Ethnicity (ref: Non-Chinese)							
Chinese	*0.67*	*0.57*	*0.91*	*0.74*	*1.04*	*0.93*	*0.53*
95% CI	*0.38; 1.19*	*0.38; 0.85*	*0.50; 1.65*	*0.40; 1.38*	*0.70; 1.56*	*0.62; 1.41*	*0.30; 0.92*
*P* value	*0.17*	*0.01*	*0.76*	*0.35*	*0.84*	*0.75*	*0.03*
Marital status (ref: Not married)							
Married	1.65	1.75	2.07	3.24	1.43	1.15	0.71
95% CI	*1.09; 2.49*	*1.26; 2.43*	*1.31; 3.27*	*2.01; 5.23*	*1.03; 1.99*	*0.82; 1.61*	*0.42; 1.20*
*P* value	*0.02*	*0.00*	*0.00*	*0.00*	*0.03*	*0.41*	*0.20*
Educational level (ref: below university level)							
University and above	2.07	2.41	1.43	1.35	1.46	1.51	1.21
95% CI	*1.41; 3.03*	*1.76; 3.30*	*0.94; 2.17*	*0.89; 2.07*	*1.08; 1.98*	*1.09; 2.08*	*0.77; 1.92*
*P* value	*0.00*	*0.00*	*0.10*	*0.16*	*0.02*	*0.01*	*0.41*
Number of chronic conditions	1.06	1.07	0.96	1.14	1.08	1.19	0.89
95% CI	*0.92; 1.23*	*0.96; 1.19*	*0.83; 1.11*	*0.95; 1.36*	*0.96; 1.20*	*1.07; 1.33*	*0.78; 1.03*
*P* value	*0.41*	*0.21*	*0.57*	*0.15*	*0.20*	*0.00*	*0.11*
Living with elderly (ref: No)							
Yes	0.95	0.98	1.47	1.40	0.89	0.77	0.91
95% CI	*0.64; 1.40*	*0.72; 1.33*	*0.96; 2.25*	*0.91; 2.16*	*0.66; 1.21*	*0.56; 1.05*	*0.57; 1.44*
*P* value	*0.79*	*0.89*	*0.08*	*0.13*	*0.45*	*0.10*	*0.68*
Constant	1.16	0.27	1.13	0.90	0.29	0.18	0.66
95% CI	*0.50; 2.70*	*0.14; 0.53*	*0.46; 2.78*	*0.35; 2.29*	*0.15; 1.58*	*0.09; 0.36*	*0.24; 1.83*
*P* value	*0.74*	*0.00*	*0.79*	*0.82*	*0.00*	*0.00*	*0.43*
Log likelihoods	-373.61	-542.61	-321.69	-314.82	-549.29	-519.00	-251.95

Abbreviations: COVID-19, coronavirus disease 2019; GAD, general anxiety disorder; ICU, intensive care unit. 95% confidence intervals presented below odds ratios in italics. * N = 426 as “Not applicable” responses for the variable “Keeping children out of school” were removed as the respondents may not have school-aged children.

####  Personal Characteristics and Adoption of Preventive Behaviours

 In relation to demographic variables that predicted the adoption of preventive behaviours during COVID-19, it is important to note that higher anxiety (GAD-7 scores) predicted higher odds of cancelling social events, avoiding public transport, wearing masks in public, avoiding hospitals or clinics or keeping children out of school.

 Additionally, respondents who were aged 21–35 compared to those >50 years old, had higher odds of wearing their masks in public. Chinese, compared to non-Chinese participants, had lower odds of avoiding public transport and keeping children out of school. Married respondents, compared to those who were not married, also reported higher odds of cancelling social events, avoiding public transport, reducing time spent shopping and eating out and wearing their masks in public. Respondents with university education or above, compared to those with below university education, had higher odds of cancelling social events, avoiding public transport, wearing masks in public and avoiding hospitals or clinics. The higher the number of chronic conditions reported by respondents, the higher their odds were of avoiding hospitals and clinics. Those living with the elderly had higher odds of reducing their time spent shopping compared to respondents not living with elderly individuals (Table 3).

###  Predictors of Preventive Behaviours That Could Be Adopted With Increased Frequency During COVID-19

####  Public Perception of COVID-19 and Preventive Behaviours That Could Be Adopted With Increased Frequency

 Respondents who thought their chances of getting COVID-19 were higher in comparison to regular influenza did not significantly predict increased frequency of any preventive behaviours. The perceived likelihood of COVID-19-related ICU admission was associated with an increased frequency of cleaning the environment. Individuals who agreed that the government would be successful in containing the spread of COVID-19 had higher of odds of increasing the frequency of washing hands. Additionally, respondents who agreed that they could reduce their chances of getting COVID-19 if they were careful, had higher odds of avoiding touching surfaces in public and working/studying from home. However, those who agreed that Singaporeans were overly concerned or paranoid regarding COVID-19 had lower odds of increasing the frequency of washing their hands and avoiding touching public surfaces.

 Similarly, response efficacy also predicted all the examined preventive behaviours that could be adopted with increased frequency (washing hands, cleaning environment, avoid touching public surfaces and working/studying from home) and had the largest odds ratios for all preventive behaviours that could be adopted with increased frequency (Table 4).

**Table 4:** Ordered Logistic Regressions on the Predictors of Preventive Behaviours that can be Adopted with Increased Frequency, N = 897 (Proportional Odds Ratios)

	**Washing Hands**	**Cleaning Environment**	**Avoid Touching Public Surfaces**	**Working/Studying From Home**
**Public Perceptions of COVID-19 in Singapore**				
Chances of getting COVID-19 compared to the flu (influenza) (ref: Same/lower)				
Higher (slightly higher, much higher)	1.22	1.20	1.12	0.99
95% CI	*0.94; 1.57*	*0.93; 1.54*	*0.86; 1.45*	*0.77; 1.28*
*P* value	*0.14*	*0.16*	*0.40*	*0.95*
Likelihood of ICU Admission if you get COVID-19 (ref: unlikely)				
Likely (very likely, somewhat likely)	1.05	1.26	1.00	1.22
95% CI	*0.81; 1.37*	*0.97; 1.64*	*0.76; 1.31*	*0.93; 1.58*
*P* value	*0.71*	*0.08*	*0.99*	*0.15*
Government Will be Successful in Containing the Spread of COVID-19 (ref: disagree/no opinion)				
Agree (Strongly agree, somewhat agree)	1.48	1.28	1.09	1.24
95% CI	*1.08; 2.03*	*0.93; 1.76*	*0.79; 1.50*	*0.91; 1.70*
*P* value	*0.02*	*0.12*	*0.62*	*0.17*
I think that if I am careful, I can reduce my chances of getting COVID-19 (ref: disagree/no opinion)				
Agree (Strongly agree, somewhat agree)	1.19	1.05	1.32	1.51
95% CI	*0.88; 1.61*	*0.78; 1.42*	*0.97; 1.80*	*1.11; 2.06*
*P* value	*0.27*	*0.75*	*0.07*	*0.01*
I think that people in Singapore are overly worried/paranoid about COVID-19 (ref: disagree/no opinion)				
Agree (Strongly agree, somewhat agree)	0.75	1.18	0.70	1.01
95% CI	*0.58; 0.98*	*0.91; 1.53*	*0.54; 0.92*	*0.77; 1.32*
*P* value	*0.03*	*0.22*	*0.01*	*0.93*
Effectiveness of strategies in reducing risk of getting the COVID-19 Virus (ref: not effective/somewhat effective)				
Very effective	2.66	2.99	4.73	2.87
95% CI	*2.02; 3.51*	*2.27; 3.94*	*3.56; 6.27*	*2.20; 3.76*
*P* value	*0.00*	*0.00*	*0.00*	*0.00*
**Participant Demographics**				
GAD (anxiety)	1.03	1.06	1.05	1.01
95% CI	*1.00; 1.05*	*1.03; 1.09*	*1.02; 1.07*	*0.99; 1.04*
*P* value	*0.05*	*0.00*	*0.00*	*0.28*
Age categories (ref: >50 years old)				
21–35 years old	0.60	0.69	0.71	1.43
95% CI	*0.41; 0.88*	*0.47; 1.00*	*0.48; 1.03*	*0.98; 2.08*
*P* value	*0.01*	*0.05*	*0.07*	*0.06*
36–50 years old	0.65	0.80	0.81	1.32
95% CI	*0.46; 0.91*	*0.57; 1.13*	*0.58; 1.15*	*0.94; 1.85*
*P* value	*0.01*	*0.21*	*0.24*	*0.11*
Ethnicity (ref: Non-Chinese)				
Chinese	*0.87*	*0.88*	*0.71*	*0.79*
95% CI	*0.61; 1.24*	*0.61; 1.25*	*0.50; 1.03*	*0.55; 1.12*
*P* value	*0.43*	*0.47*	*0.07*	*0.18*
Marital status (ref: Not married)				
Married	1.01	1.23	1.18	1.16
95% CI	*0.76; 1.34*	*0.93; 1.63*	*0.88; 1.58*	*0.87; 1.55*
*P* value	*0.95*	*0.16*	*0.26*	*0.31*
Educational level (ref: Below university level)				
University and above	1.40	1.18	1.88	2.44
95% CI	*1.06; 1.83*	*0.90; 1.55*	*1.42; 2.48*	*1.85; 3.21*
*P* value	*0.02*	*0.23*	*0.00*	*0.00*
Number of chronic conditions	1.06	1.04	1.10	1.01
95% CI	*0.96; 1.16*	*0.94; 1.14*	*0.99; 1.21*	*0.92; 1.11*
*P* value	*0.26*	*0.44*	*0.07*	*0.83*
Living with elderly (ref: No)				
Yes	0.90	0.91	0.96	0.79
95% CI	*0.69; 1.18*	*0.70; 1.19*	*0.73; 1.26*	*0.60; 1.03*
*P* value	*0.45*	*0.48*	*0.77*	*0.08*
Cut 1	-1.01	0.00	-0.43	0.97
95% CI	-1.63; -0.39	-0.60; 0.60	-1.05; 0.18	0.37; 1.58
Cut 2	1.18	2.14	1.77	2.38
95% CI	0.56; 1.80	1.53; 2.76	1.14; 2.39	1.76; 3.01
Log likelihoods	-988.52	-899.14	-841.85	-895.70

Abbreviations: COVID-19, coronavirus disease 2019; GAD, general anxiety disorder; ICU, intensive care unit. 95% confidence intervals presented below proportional odds ratio in italics.

####  Personal Characteristics and Preventive Behaviours That Could Be Adopted With Increased Frequency

 Higher scores on the GAD-7 were also predictive of increased frequency in many preventive behaviours including washing hands, cleaning environment, avoiding touching public surfaces.

 Additionally, those aged 21–35, compared to those >50, reported lower odds of washing hands, cleaning environment, avoid touching public surfaces but higher odds of working/studying from home. Participants aged 36–50, compared to those >50, also had lower odds of washing their hands. Ethnicity (Chinese vs. non-Chinese) was associated with lower odds of avoiding touching public places. Married respondents, compared to those who were not, was not associated with increased/decreased odds of increasing frequency of any preventive behaviours. Those who were university educated and above, compared to those who had below university level education, had higher odds of increasing their frequency of washing hands, avoiding touching public surfaces and working/studying from home. Number of reported chronic conditions, however, was only associated with the increased odds of avoiding touching public places. Respondents who lived with elderly individuals – compared to those who did not – had lower odds of working/studying from home (Table 4).

## Discussion

 This study aimed to examine public perceptions toward COVID-19 and to identify predictors of COVID-19 preventive behaviours in Singapore. Results suggested that though adoption of individual preventive behaviours varied, adoption of the preventive behaviours among Singaporeans was, overall, high. Respondents who perceived higher risks, government trust, believed they could decrease their risk of COVID-19, perceived appropriateness of COVID-19 behaviours, and, and had higher response efficacy reported increased adoption and/or frequency of preventive measures. Regarding respondent characteristics, older individuals, those who are educated, anxious, and married were more likely to adopt or increase frequency of preventive measures. Importantly, about 24% of the sample reported moderate (GAD-7: 10–14) or severe (GAD-7: ≥15) anxiety.

###  Adoption of Preventive Behaviours

 Consistent with government advisories restricting social interaction,^40^ the most widely adopted measures to avoid COVID-19 were avoiding or cancelling social events (82.8%) and reducing time spent shopping/eating out (86.2%/86.1%). It should be noted, however, that among these widely adopted preventive behaviours, adoption of mask wearing was not as widespread (57.5%). This could be because, unlike recommendations regarding social distancing, government advisories regarding mask wearing have not been consistent or clear. Earlier advisories suggested that mask wearing was of limited use and practicability, and only recommended for sick individuals^41,42^ – in contrast with later measures that recommended wearing masks in public.^43^ Our results demonstrated that, in accordance to past literature, recommended measures have to be consistent and clear to encourage adoption.

 Lowest adoption rates were reported for avoiding hospitals/clinics (33.8%), avoiding public transport (44%) and keeping children out of school (43.2%). There may be several reasons for these findings. It is possible that the reticence among Singaporeans regarding avoiding public transport may be due to the unclear, inconsistent information regarding the safety of utilising public transport during the COVID-19 pandemic.^44-46^ We also have to consider the essentialness of public transport in Singapore.^47^ Due to low car ownership in the country, a significant number of Singaporean families are reliant on public transport for daily, essential activities, making avoiding them unfeasible. The same argument of necessity can be applied to the low avoidance rates of hospitals/clinics – they may also be seen as unavoidable for individuals with health conditions. The choice of parents’ to keep their children in school can also be explained by earlier government reports recommending them to do so.^48,49^ These findings suggest that clear and consistent information is necessary to influence appropriate preventive behaviours.

 Regarding preventive behaviours that could be adopted with increased frequency,the majority of our respondents increased their frequency of hygienic practices (a consistently highlighted government recommendation) to reduce their risks of getting COVID-19. However, of note is a significant percentage of respondents who did not increase their frequency of working or studying from home (38.4%). This may be because work-from-home arrangements are a luxury not afforded to many essential jobs (eg, those in the service sectors, teachers). Telecommuting was also not widely implemented in Singapore prior to the COVID-19 pandemic,^50^ and adoption may be slow.

###  Predictors of Preventive Behaviours

 In support of our hypotheses, perceived risks related to COVID-19 were found to be associated with the adoption of some preventive behaviours. These findings can be understood within the context of PMT, which states – broadly – that the higher the perceived risks and vulnerability felt by the public, the higher the adoption of preventive behaviours.^51,52^ However, many preventive behaviours in this study were *not* predicted by perceived risks. This discrepancy may be due to confusions regarding the severity of COVID-19 risk. Specifically, there is – at present – a lack of scientific consensus across geological regions regarding the risk and severity of the virus.^53-55^ This, combined with misinformation prevalent in social media sites,^56^ may have precipitated large divides among the population regarding risk and severity of COVID-19. In support of our working hypothesis, results indicate significant discrepancies among respondents, with 55% perceiving their risks of contracting COVID-19 to be lower or the same as the normal flu (influenza). These results may have several important policy implications. First, they suggest that greater efforts may be required to combat misinformation. Second, they reinforce the argument that clear and consistent information throughout the pandemic is necessary for the public to motivate appropriate preventive behaviours.

 As expected,^23^ results indicated that government trust predicted increased frequency of some preventive measures. A potential explanation for this finding may be that when governments are viewed as trustworthy, their measures are also seen as dependable, which in turn encourages the adoption of their recommendations. It should be noted, however, that government trust did not consistently predict the adoption and increased frequency of many preventive behaviours. While unexpected, this may be due to high baseline level of governmental trust already prevalent among Singaporeans.^57,58^ Nonetheless, these findings underline the importance of government trust toward ensuring public adoption of the preventive measures.

 Supporting our hypothesis, individuals with higher levels of self-efficacy were more likely to adopt and increase the frequency of their preventive behaviours. This association between self-efficacy (ie, I can decrease my risk of infection) and adoption of preventive behaviours has been consistently underlined in literature,^17,18^ suggesting that the promotion of self-efficacy should be an important factor of consideration for educational interventions and public health messages.

 Consistent with our hypotheses, response efficacy was also found to be an important predictor of preventive behaviours – response efficacy predicted the adoption of *all* the respective preventive behaviours. It also had the largest odds ratios indicating that response efficacy had the strongest association with adoption of preventive behaviours. These findings are expected, and support PMT, which suggests that when faced with a threat to health (ie, COVID-19), the level of response efficacy of the preventive measure directly predicts its adoption.^59,60^ These strong findings are of particular consequence as they demonstrate that the response efficacy play an essential role in increasing the adoption and frequency of the preventive measures. For increased public adoption of preventive measures, educational programs and public health messages must focus on the effectiveness of their preventive measures.

 A quarter of the sample reported moderate to severe anxiety which warrants for further assessment and referral to a mental health specialist. These results suggest that anxiety could be a potential problem among general population during a large pandemic like COVID-19. Comparable to extant literature, we found that anxiety, older age, higher education, and marital status predicted the adoption and frequency of some preventive behaviours. However, given their particular vulnerability to COVID-19, the lack of associations between number of chronic conditions and preventive behaviours must be noted. Our results underline segments of population that could be targeted by tailored public health messages to promote the adoption of preventive measures.

###  Significance and Implications

 Overall, the main strength of this study was its ability to pinpoint the significant drivers of behaviour adoption during a large-scale pandemic of unprecedented severity. It should also be noted that while this study was conducted in Singapore, its findings may also be generalizable to other Asian countries with similar societal, cultural and governmental structures (ie, countries with obedient, collectivistic populations such as Japan, China, etc).^61^ Our findings serve to increase governmental preparedness for future pandemics and also to provide important lessons for most countries in designing public health messages directed towards increasing the public adoption of preventive measures.

###  Study Limitations and Future Research

 Our findings have to be taken in the context of several limitations. First, our findings may not be generalizable to individualistic societies. For example, studies from individualistic societies compared to the results from our study show distinctively lower adoption rates of many preventive behaviours.^62,63^ While examining the impact that culture may have on the adoption of preventive measures is beyond the scope of this study, future studies may consider a multi-country sample with participants recruited across different cultural regions. Additionally, no reliability analyses were conducted on the author-designed questions – it is possible that responses may not correlate with actual behaviour. Future studies should include (if possible) validated measures of COVID-19 perceptions. Finally, our sample overrepresented highly educated respondents (only 12.3% of our respondents reported not having at least college-level education (Institute of Technical Education; ITE and below). This is consistent with most web-enabled surveys as they tend to attract individuals with higher education. However, conducting an online survey enabled us to collect time-sensitive information much faster than other methods.

## Conclusion

 By examining public perception regarding COVID-19 and the predictors of preventive behaviours adopted by the Singaporean public, we found that to influence appropriate preventive behaviours, preventive measures have to be consistent, accurate, and clear. Additionally, to ensure consistent adoption of preventive measures, government programs should focus on promoting trust in government response, self-efficacy, and underline the effectiveness of respective preventive behaviours. Particular focus should be on demographic segments with likely low adoption, such as younger individuals and those with lower education. The study findings are expected to aid in increasing governmental preparedness for future outbreaks and help in developing educational programs and designing public health messages directed at increasing the adoption of preventive behaviours.

## Acknowledgements

 We would like to thank Dr. Chetna Malhotra and Dr. Irene Teo for their comments on the survey instrument.

## Ethical issues

 This study was exempted from review by the National University of Singapore Institutional Review Board (Application Reference Number: S-20-085).

## Competing interests

 Authors declare that they have no competing interests.

## Authors’ contributions

 SO: Conceptualisation and design of study, data analysis, drafting of manuscript. SNYW: Drafting of manuscript (Introduction, methods, results, discussion). IC: Data analyses, and review and revision of manuscript. EAF: Review and revision of manuscript.

## Funding

 This project is funded by Lien Centre for Palliative Care (LCPC Research N-911-000-030-091), Duke-NUS Medical School.
